# Detection of nasopharyngeal carcinoma susceptibility with single nucleotide polymorphism analysis using next-generation sequencing technology

**DOI:** 10.18632/oncotarget.17085

**Published:** 2017-04-13

**Authors:** Mu-Yun Wu, Shu-Jing Huang, Fan Yang, Xin-Tian Qin, Dong Liu, Ying Ding, Shu Yang, Xi-Cheng Wang

**Affiliations:** ^1^ Department of Oncology, The First Affiliated Hospital of Guangdong Pharmaceutical University, Guangzhou, China; ^2^ Department of Oncology, The Fifth People's Hospital of Wuhu, Wuhu, China

**Keywords:** nasopharyngeal carcinoma (NPC), next-generation sequencing technology (NGS), susceptibility, single nucleotide polymorphism (SNP)

## Abstract

Nasopharyngeal carcinoma (NPC) is a head and neck cancer with high incidence in South China and East Asia. To provide a theoretical basis for NPC risk screening and early prevention, we conducted a meta-analysis of relevant literature on the association of single nucleotide polymorphisms (SNP)s with NPC susceptibility. Further, expression of 15 candidate SNPs identified in the meta-analysis was evaluated in a cohort of NPC patients and healthy volunteers using next-generation sequencing technology. Among the 15 SNPs detected in the meta-analysis, miR-146a (rs2910164, C>G), HCG9 (rs3869062, A>G), HCG9 (rs16896923, T>C), MMP2 (rs243865, C>T), GABBR1 (rs2076483, T>C), and TP53 (rs1042522, C>G) were associated with decreased susceptibility to NPC, while GSTM1 (+/DEL), IL-10 (rs1800896, A>G), MDM2 (rs2279744, T>G), MDS1-EVI1 (rs6774494, G>A), XPC (rs2228000, C>T), HLA-F (rs3129055, T>C), SPLUNC1 (rs2752903, T>C; and rs750064, A>G), and GABBR1 (rs29232, G>A) were associated with increased susceptibility to NPC. In our case-control study, an association with increased risk for NPC was found for the AG vs AA genotype in HCG9 (rs3869062, A>G). In addition, heterozygous deletion of the GSTM1 allele was associated with increased susceptibility to NPC, while an SNP in GABBR1 (rs29232, G>A) was associated with decreased risk, and might thus have a protective role on NPC carcinogenesis. This work provides the first comprehensive assessment of SNP expression and its relationship to NPC risk. It suggests the need for well-designed, larger confirmatory studies to validate its findings.

## INTRODUCTION

Nasopharyngeal carcinoma (NPC) is a type of head and neck neoplasm typically associated with cervical lymphadenopathy, epistaxis, and rhinocleisis, that shows high incidence in South China and East Asia. Although the exact cause of NPC is in most cases unclear, environmental factors, certain dietary factors, and Epstein-Barr virus (EBV) infection are known to be closely related with its development. In addition, it is believed that genetic factors are important determinants of NPC risk [1–3].

A single nucleotide polymorphism (SNP) is a third-generation genetic marker, after first-generation restriction fragment length polymorphism and second-generation simple sequence length (microsatellite) polymorphism. A SNP is a polymorphism in a gene's sequence caused by a variation in a single nucleotide. Research on SNPs focus mainly on their impact on three classes of genes: tumor suppressor genes, metabolic enzymes genes, and DNA repair genes, and is an important new approach to understand disease susceptibility and pathogenesis, and to guide diagnosis and individual treatment choice.

SNPs are usually detected by polymerase chain reaction (PCR), which is a technology with low speed and efficiency. The introduction of next-generation sequencing technology (NGS), however, made it possible the detection of SNPs with high-throughput. NGS, used to sequence hundreds of thousands to millions of genetic molecules in parallel, is a second-generation sequencing technology developed after first generation (Sanger) sequencing. NGS is much more convenient than the traditional genetic testing technologies [4, 5]. At present, NGS is mainly used in the analysis of whole-genome gene expression profiles, tumor genome re-sequencing, whole-genome small molecule RNA analysis, whole-genome methylation analysis, and chromosome structure analysis, among others [6–10].

Although many studies have focused on the detection of tumor SNPs by NGS, for example in colorectal cancer, renal cell carcinoma, ovarian cancer, etc. [11–13], there are no reports, to our knowledge, evaluating the association of SNPs with NPC susceptibility using NGS. Therefore, we conducted both a meta-analysis of the association of SNPs with NPC risk in East Asian populations, as well as a case-control study to assess the expression of the identified SNPs using NGS. Our aim was to obtain concise, reliable data on the association of SNPs with NPC susceptibility, as to provide a theoretical basis for NPC screening and help guide early prevention efforts in high-risk populations. In addition, we hope our findings will provide new strategies and ideas to improve the diagnosis and treatment of NPC.

## RESULTS

### Meta-analysis

3754 relevant articles were initially retrieved, and 1821 articles remained after eliminating duplicates. After screening the titles and abstracts according to the inclusion criteria, 184 articles were selected. Data from 97 articles were entered after full-text review. From these, we found a relationship between 279 polymorphic loci in 126 genes and susceptibility to NPC.

After eliminating polymorphic loci represented in less than two articles, 15 SNPs were found to be significantly associated with the risk of NPC: TP53 (rs1042522, C>G) [14–18], GSTM1 (+/DEL) [19–21], IL-10 (rs1800896, A>G) [22, 23], GABBR1 (rs2076483, T>C; rs29232, G>A) [24, 25], MDM2 (rs2279744, T>G) [16, 26], miR-146a (rs2910164, C>G) [27, 28], MDS1-EVI1 (rs6774494, G>A) [29, 30], XPC (rs2228000, C>T) [30, 31],, HCG9 (rs3869062, A>G; rs16896923, T>C) [24, 25], HLA-F (rs3129055, T>C) [24, 25], MMP2 (rs243865, C>T) [32, 33], SPLUNC1 (rs2752903, T>C; rs750064, A>G) [34, 35]. Among these, two SNPs (in the TP53 and GSTM1 genes) were addressed in more than two relevant articles, while each of the others were mentioned in two studies. Among the 15 SNPs identified, those located on miR-146a (rs2910164, C>G), HCG9 (rs3869062, A>G), HCG9 (rs16896923, T>C), MMP2 (rs243865, C>T), GABBR1 (rs2076483, T>C), and TP53 (rs1042522, C>G) were associated with decreased susceptibility to NPC, while the remaining SNPs were associated with an increased risk. The specific features of the 15 SNPs are shown in Table 1. The predominant characteristics and the quality scores of the eligible studies are shown in Supplementary Tables 15.

**Table 1:** Features of the 15 SNPs significantly associated with NPC

Gene	rs	Allele	Sample	Model	Summary OR (95% CI)	Studies n.
TP53	rs1042522	G	1419/1707	Fixed-effects	0.812(0.734, 0.897)	5
GSTM1	——	NULL	837/1299	Fixed-effects	1.323(1.119, 1.565)	3
IL-10	rs1800896	G	374/732	Fixed-effects	2.102(1.64,2.694)	2
GABBR1	rs2076483	C	1498/2446	Fixed-effects	0.6(0.535,0.674)	2
MDM2	rs2279744	G	1325/1475	Random-effects	1.461(1.091,1.957)	2
miR-146a	rs2910164	G	389/3976	Fixed-effects	0.714(0.603,0.847)	2
MDS1-EVI1	rs6774494	A	6267/6129	Fixed-effects	1.179(1.107,1.255)	2
XPC	rs2228000	T	1330/1340	Fixed-effects	1.196(1.1072,1.335)	2
GABBR1	rs29232	A	1498/2442	Fixed-effects	1.708(1.556, 1.875)	2
HCG9	rs3869062	G	1484/2432	Fixed-effects	0.566(0.508, 0.631)	2
HLA-F	rs3129055	C	1500/2445	Fixed-effects	1.152(1.373, 1.665)	2
HCG9	rs16896923	C	1494/2439	Fixed-effects	0.575(0.509, 0.649)	2
MMP2	rs243865	T	1173/1149	Fixed-effects	0.632(0.492,0.811)	2
SPLUNC1	rs2752903	C	684/768	Fixed-effects	1.764(1.408,2.21)	2
SPLUNC1	rs750064	G	313/429	Fixed-effects	1.462(1.184,1.804)	2

### Case-control study

**Figure 1:**
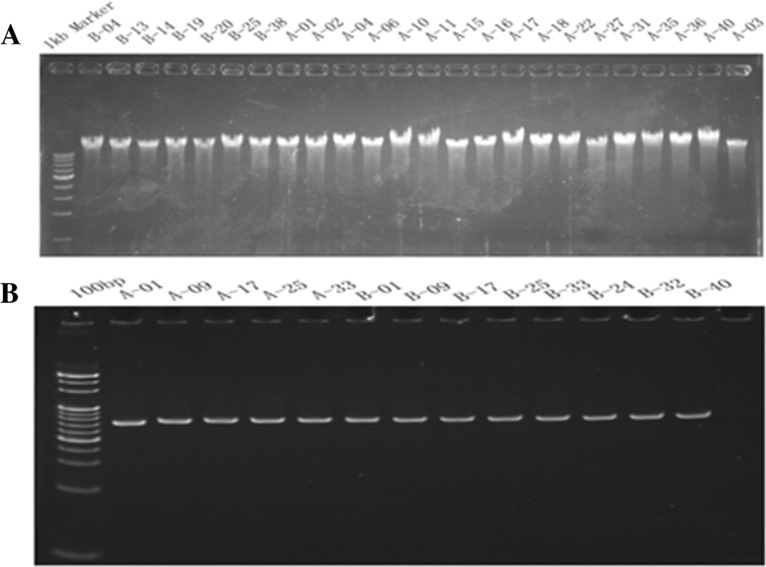
Gene quality testing electrophoretogram **(A)** DNA agarose gel electrophoresis detection; **(B)** Target fragments detection by PAGE gel electrophoresis.

Peripheral venous blood samples from 40 NPC patients and 40 healthy volunteers in South China were collected. There were 33 men and 7 women in the case group (Group A), in which the age distribution (mean ± standard deviation) was 46.05 ± 13.77 years. There were 15 men and 25 women in the control group (Group B), with an age distribution (mean ± standard deviation) of 36.13 ± 10.20 years. The results of the DNA and target fragments’ quality testing are shown in Figure 1. A single, clear main band and few number of side band indicated that the quality of the extracted DNA and the amplified target fragments was high.

**Figure 2:**
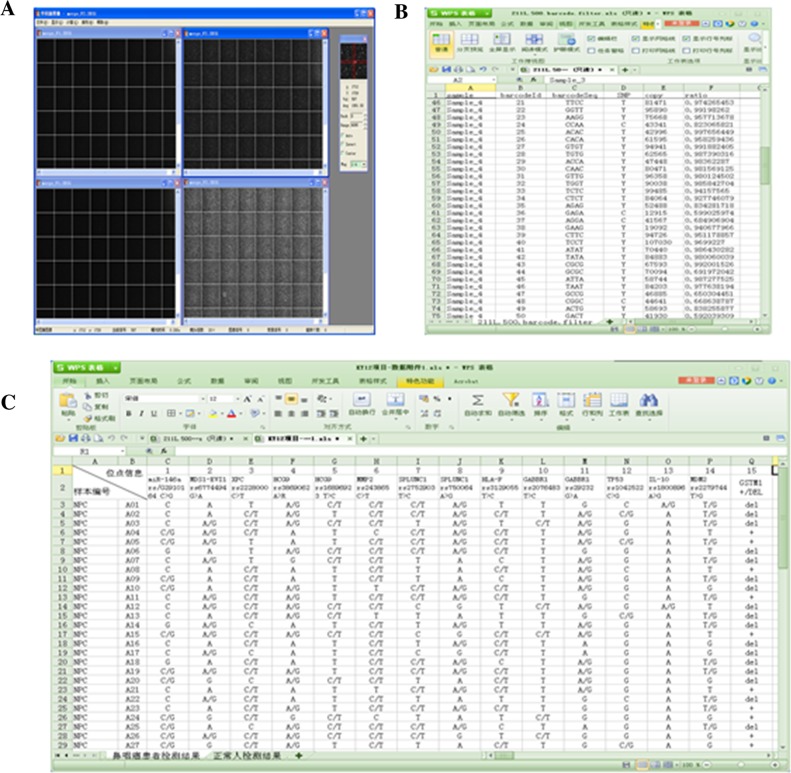
Automatic detection and analysis with the PSTAR high-throughput instrument **(A)** High-throughput sequence image, base recognition and data processing; **(B)** High-throughput sequencing results file generation; **(C)** High-throughput sequencing results statistics.

Partial data of automatic sequence, base recognition and data processing by the P-STAR high-throughput instrument are shown in Figure 2. The 15 SNP loci genotypes of all 80 samples could be obtained through automatic analysis with the PSTAR sequencer.

The specific gene distribution of the 15 SNPs in the case group (Group A) and the control group (Group B) is shown in Table 2 and Table 3. SNP-15 (GSTM1) could not be subjected to Hardy-Weinberg equilibrium (HWE) test because it has only two genotypes. Thus, the HWE test was performed on the case and control groups for the other 14 SNPs, and the results are shown in Table 4.

**Table 2:** Specific gene distribution of identified SNPs, except SNP-15 (GSMT1) which has only two genotypes

All Samples		Group A (40)		Group B (40)	
SNP	Genotype	Sample Counts		Sample Counts	
**SNP-01**					
miR-146a	CC	16	40.00%	16	40.00%
rs2910164	GG	4	10.00%	6	15.00%
C>G	CG	20	50.00%	18	45.00%
**SNP-02**					
MDS1-EVI1	GG	5	12.50%	6	15.00%
rs6774494	AA	18	45.00%	13	32.50%
G>A	GA	17	42.50%	21	52.50%
**SNP-03**					
XPC	CC	6	15.00%	11	27.50%
rs2228000	TT	5	12.50%	3	7.50%
C>T	CT	29	72.50%	26	65.00%
**SNP-04**					
HCG9	AA	15	37.50%	7	17.50%
rs3869062	GG	4	10.00%	3	7.50%
A>G	AG	21	52.50%	30	75.00%
**SNP-05**					
HCG9	TT	25	62.50%	22	55.00%
rs16896923	CC	0	0.00%	0	0.00%
T>C	TC	15	37.50%	18	45.00%
**SNP-06**					
MMP2	CC	2	5.00%	0	0.00%
rs243865	TT	6	15.00%	8	20.00%
C>T	CT	32	80.00%	32	80.00%
**SNP-07**					
SPLUNC1	TT	20	50.00%	21	52.50%
rs2752903	CC	3	7.50%	0	0.00%
T>C	TC	17	42.50%	19	47.50%
**SNP-08**					
SPLUNC1	AA	13	32.50%	10	25.00%
rs750064	GG	5	12.50%	7	17.50%
A>G	AG	22	55.00%	23	57.50%
**SNP-09**					
HLA-F	TT	14	35.00%	18	45.00%
rs3129055	CC	5	12.50%	2	5.00%
T>C	CT	21	52.50%	20	50.00%
**SNP-10**					
GABBR1	TT	29	72.50%	24	60.00%
rs2076483	CC	0	0.00%	0	0.00%
T>C	CT	11	27.50%	16	40.00%
**SNP-11**					
GABBR1	GG	11	27.50%	26	65.00%
rs29232	AA	6	15.00%	0	0.00%
G>A	GA	23	57.50%	14	35.00%
**SNP-12**					
TP53	CC	6	15.00%	8	20.00%
rs1042522	GG	29	72.50%	20	50.00%
C>G	CG	5	12.50%	12	30.00%
**SNP-13**					
IL-10	AA	37	92.50%	37	92.50%
rs1800896	GG	0	0.00%	0	0.00%
A>G	AG	3	7.50%	3	7.50%
**SNP-14**					
MDM2	TT	9	22.50%	8	20.00%
rs2279744	GG	9	22.50%	10	25.00%
T>G	TG	22	55.00%	22	55.00%

**Table 3:** Distribution of the 15 SNP alleles

All Allele		Group A (80)		Group B (80)	
SNP	Allele	Counts		Counts	
**SNP-01**					
miR-146a	C	52	0.65	50	0.625
rs2910164	G	28	0.35	30	0.375
**SNP-02**					
MDS1-EVI1	G	27	33.75%	33	41.25%
rs6774494	A	53	66.25%	47	58.75%
**SNP-03**					
XPC	C	41	51.25%	48	60.00%
rs2228000	T	39	48.75%	32	40.00%
**SNP-04**					
HCG9	A	51	63.75%	44	55.00%
rs3869062	G	29	36.25%	36	45.00%
**SNP-05**					
HCG9	T	65	81.25%	62	77.50%
rs16896923	C	15	18.75%	18	22.50%
**SNP-06**					
MMP2	C	36	45.00%	32	40.00%
rs243865	T	44	55.00%	48	60.00%
**SNP-07**					
SPLUNC1	T	57	71.25%	61	76.25%
rs2752903	C	23	28.75%	19	23.75%
**SNP-08**					
SPLUNC1	A	48	60.00%	43	53.75%
rs750064	G	32	40.00%	37	46.25%
**SNP-09**					
HLA-F	T	49	61.25%	56	70.00%
rs3129055	C	31	38.75%	24	30.00%
**SNP-10**					
GABBR1	T	69	86.25%	64	80.00%
rs2076483	C	11	13.75%	16	20.00%
**SNP-11**					
GABBR1	G	45	56.25%	66	82.50%
rs29232	A	35	43.75%	14	17.50%
**SNP-12**					
TP53	C	17	21.25%	28	35.00%
rs1042522	G	63	78.75%	52	65.00%
**SNP-13**					
IL-10	A	77	96.25%	77	96.25%
rs1800896	G	3	3.75%	3	3.75%
**SNP-14**					
MDM2	T	40	50.00%	38	47.50%
rs2279744	G	40	50.00%	42	52.50%
**SNP-15**					
GSTM1	+	15	37.50%	25	62.50%
	DEL	25	62.50%	15	37.50%

**Table 4:** HWE test of the case group (Group A) and control group (Group B)

SNP No.	SNP	HWE (P value)	
		Group A	Group B
SNP-01	miR-146a	0.391(0.532)	0.064(0.800)
SNP-02	MDS1-EVI1	0.098(0.754)	0.277(0.599)
SNP-03	XPC	8.133(0.004)	5.017(0.025)
SNP-04	HCG9	0.739(0.390)	10.615(0.001)
SNP-05	HCG9	2.130(0.144)	3.371(0.066)
SNP-06	MMP2	15.186(0.000)	17.778(0.000)
SNP-07	SPLUNC1	0.056(0.813)	3.881(0.049)
SNP-08	SPLUNC1	0.851(0.356)	0.980(0.322)
SNP-09	HLA-F	0.449(0.503)	1.451(0.228)
SNP-10	GABBR1	1.017(0.313)	2.500(0.114)
SNP-11	GABBR1	1.132(0.287)	1.800(0.180)
SNP-12	TP53	15.701(0.000)	4.642(0.031)
SNP-13	IL-10	0.061(0.805)	0.061(0.805)
SNP-14	MDM2	0.400(0.527)	0.422(0.516)
SNP-15	GSTM1	-	-

Statistical analysis on all SNPs were performed using the homozygous wild-type genotype as reference to assess the relationship between SNPs and NPC susceptibility (Table 5). The statistical analysis yielded the following results. Firstly, the mutant heterozygous type of SNP-04 (HCG9, rs3869062, A>G) might be related to the incidence of NPC, as an increased risk was found for the AG versus the AA genotype (OR = 3.061, 95%CI: 1.064-8.804), while no association was found between the GG genotype and NPC risk. Secondly, the SNP-11 variation (GABBR1, rs29232, G>A) might have a protective role in the development of NPC, because the risk of the SNP-11 mutant genotype was lower than that of the wild-type (OR = 0.204, 95% CI: 0.079-0.528). Thirdly, genetic deletion of SNP-15 (GSTM1) might contribute to increased susceptibility to NPC. Finally, no associations were found between the remaining SNPs and NPC risk in our case-control study.

**Table 5:** Statistical analysis of the relationship between theSNPs and NPC susceptibility

	Group A	Group B	P value	OR	95%CI
**SNP-01**					
GG/CG	24	24	1.000	1.000	0.409-2.446
GG	4	6	0.826	0.900	0.351-2.307
CG	20	18	0.849	1.500	0.355-6.347
CC	16	16			
**SNP-02**					
GA/AA	35	34	0.745	0.810	0.226-2.903
AA	18	13	0.712	0.602	0.151-2.404
GA	17	21	1.000	1.029	0.267-3.963
GG	5	6			
**SNP-03**					
TT/CT	34	39	0.172	0.465	0.153-1.413
TT	5	3	0.389	0.327	0.057-1.870
CT	29	26	0.209	0.489	0.158-1.509
CC	6	11			
**SNP-04**					
GG/AG	25	33	0.045	2.829	1.003-7.977
GG	4	3	0.665	1.607	0.281-9.204
AG	21	30	0.034	3.061	1.064-8.804
AA	15	7			
**SNP-05**					
CC/TC	15	18	0.496	1.364	0.558-3.331
CC	0	0			
TC	15	18			
TT	25	22			
**SNP-06**					
TT	6	8	0.556	1.417	0.443-4.534
CT/CC	34	32			
CT	32	32			
CC	2	0			
**SNP-07**					
CC/TC	20	19	0.823	0.905	0.376-2.175
CC	3	0	0.234		
TC	17	19	0.891	1.064	0.434-2.608
TT	20	21			
**SNP-08**					
GG/AG	27	30	0.459	1.444	0.545-3.828
GG	5	7	0.489	1.820	0.443-7.477
AG	22	23	0.551	1.359	0.495-3.734
AA	13	10			
**SNP-09**					
CC/CT	26	22	0.361	0.658	0.268-1.619
CC	5	2	0.235	0.311	0.052-1.849
CT	21	20	0.526	0.741	0.293-1.875
TT	14	18			
**SNP-10**					
CC/CT	11	16	0.237	1.758	0.687-4.495
CC	0	0			
CT	11	16			
TT	29	24			
**SNP-11**					
AA/GA	29	14	0.001	0.204	0.079-0.528
AA	6	0			
GA	23	14	0.005	0.258	0.098-0.678
GG	11	26			
**SNP-12**					
GG/CG	34	32	0.556	0.706	0.221-2.259
GG	29	20	0.278	0.517	0.155-1.721
CG	5	12	0.477	1.800	0.407-7.957
CC	6	8			
**SNP-13**					
GG/AG	3	3	1.000	1.000	0.189-5.280
GG	0	0			
AG	3	3			
AA	37	37			
**SNP-14**					
GG/TG	31	32	0.785	1.161	0.397-3.395
GG	9	10	1.000	1.250	0.337-4.636
TG	22	22	0.837	1.125	0.367-3.451
TT	9	8			
**SNP-15**					
+	15	25	0.025	0.360	0.146-0.890
DEL	25	15			

The statistical analysis was made between TT gene model and CT gene model combined with CC gene model of SNP-06. The OR value of the CC gene model of SNP-07 could not be calculated because of the little number. OR, odds ratio; CI, confidence intervals.

## DISCUSSION

The study of the association of SNPs with tumor development and progression has become a hot area of research. For instance, using NGS, Gerlinger et al. found that VHL gene aberrations and an increased proportion of C>T transitions at CpG sites were closely related with the occurrence and development of clear cell renal carcinoma [12]. Stemke-Hale et al., on the other hand, reported that several SNPs and other mutations on multiple gene loci, such as KRAS c.35G>A p.Gly12Asp, BRAF V600E, and PIK3CA H1047Y, were related to borderline ovarian tumors [13]. In addition, NGS-based studies describing the association of SNPs and genetic mutations with colorectal cancer [11], breast cancer [36], glioblastoma [37], and adenoid cystic carcinoma [6], have been recently published. Although numerous studies investigated the relationship of SNPs with NPC susceptibility, the results are generally underpowered and somewhat controversial. Although many methods exist to study SNPs, such as PCR and probe hybridization technologies, only a few studies have analyzed the association of SNPs with NPC susceptibility by high-throughput sequencing technologies, especially NGS. Therefore, and to provide a theoretical basis for NPC screening and early prevention, we performed a meta-analysis to review existing SNP/NPC association data in East Asian populations, and carried out a case-control study to directly assess, using NGS, the expression of candidate SNPs in 40 NPC patients.

Our meta-analysis revealed that 15 SNPs were associated with the risk of NPC: TP53 (rs1042522, C>G), GSTM1 (+/DEL), IL-10 (rs1800896, A>G), GABBR1 (rs2076483, T>C; rs29232, G>A), MDM2 (rs2279744, T>G), miR-146a (rs2910164, C>G), MDS1-EVI1 (rs6774494, G>A), XPC (rs2228000, C>T), HCG9 (rs3869062, A>G; rs16896923, T>C), HLA-F (rs3129055, T>C), MMP2 (rs243865, C>T), and SPLUNC1 (rs2752903, T>C; rs750064, A>G). TP53 is a tumor suppressor gene located on human chromosome 17p13, and plays an important role in the stress response to hypoxia, gene damage, oncogene activation, and other injuries. TP53 also contributes to maintaining gene stability and regulating cell cycle [14–18]. Glutathione S-transferase M1 (GSTM1), a member of the glutathione s-transferase family, mediates cellular detoxification of electrophilic compounds, including carcinogens, by conjugation with glutathione [19–21]. The interleukin-10 (IL-10) gene is located on chromosome 1q31-32, and is mainly expressed in macrophages, mononuclear cells, activated B cells, and Th2 cells. IL-10 is a multifunctional negative regulatory factor and promotes tumor evasion of the immune response by downregulating the production of interferon-γ [22, 23]. The gamma-aminobutyric acid type B receptor subunit 1 gene (GABBR1), located on chromosome 6p21.3, encodes the GABAB1 receptor of gamma-aminobutyric acid, the main inhibitory neurotransmitter in the central nervous system. GABBR1 is a metabolic pathway gene, mainly related to neural network remodeling and inhibition of the diffusion of abnormal brain electrical activity [24, 25]. The murine double minute 2 (MDM2) proto-oncogene is located on chromosome 12q13-14. The MDM2 T309G polymorphism (rs2279744) can enhance MDM2 binding to the transcription factor SP1, thereby enhancing its own transcription and increasing the inhibition of the tumor suppressor TP53. MDM2 can also mediate TP53-independent tumorigenicity and control cell growth and proliferation through regulation of E2F1 expression via the MDM2-TP53-p21 signaling pathway [16, 26]. The micro ribonucleic acid-146a (miR-146a) gene, located on chromosome 5q33.3, is overexpressed or underexpressed in some tumors and functions as an oncogene or tumor suppressor gene [27, 28]. The Myelodysplastic Syndrome 1 / Ecotropic Viral Integration site-1 (MDS1-EVI1) complex locus encodes three kinds of proteins, namely EVI1, MDS1, and the fusion protein MDS1-EVI1. MDS1-EVI1 is a strong activator of promoters containing a GATA motif [29, 30]. Xeroderma pigmentosum group C (XPC), located on chromosome 3p25, is one of the key genes of the nucleotide excision repair (NER) pathway in the gene damage repair system. The main function of XPC is to identify and excise damaged sites in the genes [30, 31]. HCG9, the human leukocyte antigen (HLA) complex 9 gene, belongs to the HLA complex family along with HLA-F. The HLA complex, located on chromosome 6p21.3, is composed of a group of closely linked genes and is the most complex gene system in the human genome [24, 25]. Matrix metalloproteinase 2 (MMP2) is the main and the most widely distributed member of the matrix metalloproteinase family. It is involved in the degradation of extracellular matrix, and can destroy tissue barriers during the process of tumor cell invasion [32, 33]. The short palate lung and nasal epithelium clone 1 (SPLUNC1) gene belongs to the palate lung and nasal epithelium clone family, which has host defense functions. This gene family is expressed mostly on the epithelial surface of the respiratory and digestive tracts and play a role in signal transduction of external stimuli. SPLUNC1 is a natural immune protective molecule; it has anti-inflammatory actions, tumor suppressor effects, and helps maintain the normal physiology of the upper respiratory tract [34, 35]. Our analysis indicates that miR-146a (rs2910164, C>G), HCG9 (rs3869062, A>G), HCG9 (rs16896923, T>C), MMP2 (rs243865, C>T), GABBR1 (rs2076483, T>C), and TP53 (rs1042522, C>G) are associated with decreased susceptibility to NPC, while the other SNPs might contribute instead to increased risk of NPC. Based on our meta-analysis’ results, we carried out a case-control study to evaluate, using NGS, the presence of the 15 candidate SNPs in NPC patients and healthy controls. To this end, blood samples were extracted and amplified to obtain a large number of target genes, which were then sequenced by the PSTAR high-throughput instrument. We found a significantly increased risk for NPC in individuals carrying the AG genotype of HCG9 (rs3869062, A>G), as compared with those carrying AA alleles. A positive association with NPC was also detected for a genetic deletion (i.e. heterozygous genotype) of the GSTM1 allele. In contrast, our study indicated a negative association between a SNP in GABBR1 (rs29232, G>A) and NPC, which might indicate a protective role for this gene variant in the carcinogenesis of NPC. In contrast with most previous findings, no associations were found between the remaining candidate SNPs and NPC risk.

In our case-control study, the positive association of HCG9 (rs3869062) and GABBR1 (rs29232) with NPC risk contrasts with the negative association detected between these SNPs and NPC risk in our meta-analysis. The reason of this discrepancy may be that the sample size of our case-control experiment is small, and statistical analysis could not be carried out for some genotypes. Other limitations of our case-control study is that no consideration was paid to possible gene interactions, likely to affect the development of NPC and other cancers as well. However, our study identified three SNPs which are most likely related to NPC susceptibility, and provides a basis for further SNP analyses of NPC risk.

Hundreds of thousands to millions of DNA molecules can be analyzed in parallel using NGS technology, making gene analysis much more convenient and quick. Therefore, our future research plan is to enlarge the sample size to confirm through NGS the association of the three significant genetic variants [HCG9 (rs3869062), GABBR1 (rs29232), and GSTM1 (^+^/DEL)] with NPC susceptibility. This should allow validation of a robust NPC risk screening protocol useful to detect at-risk population and guide early prevention efforts.

## MATERIALS AND METHODS

### Meta-analysis methods and SNP identification

A comprehensive search strategy was performed to identify relevant case-control studies about the association of SNPs with NPC susceptibility. Using electronic databases, including PubMed (http://www.ncbi.nlm.nih.gov/pubmed/), Web of Science (http://wokinfo.com/) and SD (http://www.science-direct.com/), a search strategy was performed based on combinations of the keywords ‘nasopharyngeal, rhinopharyngeal, nasopharynx, rhinopharynx, or nasal part of pharynx’ and ‘cancer, tumor, tumour, neoplasm, carcinoma or adenocarcinoma’ and ‘polymorphism, variation, genetic, mutation, variant, or SNP’. The last search was updated on April 1, 2014. Although no language restrictions were applied initially, for the full-text review and final analysis the resources only permitted the review of studies published in English. The following eligibility criteria were used: i) studies published in English; ii) case-control design; iii) evaluating the association between SNP and NPC susceptibility; iv) participants were from East Asia, including China, Japan and Korea; and v) the studies indicated sample size, distribution of alleles, genotypes, or other information which could aid in inferring the estimation of odds ratios (OR) and their 95% confidence intervals (CI). Data from the eligible studies, selected in strict accordance with the inclusion criteria, were independently extracted by two investigators. Controversial issues were resolved following group discussion. The following data were extracted from each study: first author's name, publication year, gene, SNP, rs number, country of origin, genotyping method, conclusion (positive/negative), total number of cases and controls, genotype or allele distribution, and the P-value for Hardy-Weinberg equilibrium (HWE). The quality of the eligible studies was assessed by two investigators independently, according to a set of predefined criteria (Supplementary Table 16) originally proposed by Thankkinstian et al [38]. The revised criteria cover the representativeness of cases, the credibility of controls, specimens of cases determining genotypes, HWE in the controls, and total sample size. Disagreements between investigators were resolved by consensus. Study quality scores ranged between 0 (lowest) and 15 (highest). Studies with scores ≥10 were considered high-quality, while those with scores <10 were considered low-quality studies. Articles addressing the same SNP(s) were combined for meta-analysis. Summary ORs and corresponding 95% CIs were used to estimate the strength of the associations between each SNP and NPC risk in different comparison models. Z test was used to evaluate the statistical significance of pooled OR values. The statistical heterogeneity among studies was assessed by Q test and I^2^ statistics. A random-effects model (DerSimonian and Laird method) was used to estimate the summary OR when the result of the Q test was P < 0.1 or I^2^ ≥ 50%, which indicated the presence of heterogeneity. The fixed-effects model (Mantel-Haenszel method) was used when the result of the Q test was P ≥ 0.1 and I^2^ < 50%, which indicated the absence of heterogeneity. Stata software, version 11.0 (Stata Corp., College Station, TX, USA) was used for all statistical analyses. All P-values were two-sided.

### Case-study

#### Subjects recruitment

The *case group* consisted of 40 subjects from South China that were pathologically diagnosed with NPC and treated at the Department of Oncology of the First Affiliated Hospital of Guangdong Pharmaceutical University between June 2014 and July 2014. The *control group* consisted of 40 healthy people from South China that underwent physical examination in the Medical Center of the same hospital during the same time. All subjects signed informed consent. This study was approved by the ethics committees of the First Affiliated Hospital of Guangdong Pharmaceutical University.

### SNP genotyping

A 5 ml venous blood sample was collected from each subject. The samples from the 40 NPC patients in the case group (Group A), were numbered A01 to A40. Likewise, the 40 blood samples from the healthy volunteers in the control group (Group B), were numbered B01 to B40. Numbering and basic information of the 15 SNPs associated with NPC susceptibility, as detected by meta-analysis, are shown in Table 6. DNA was extracted from blood samples using the HYK Blood DNA mini Extraction Kit (HYK, Shenzhen, China). The quality and concentration of genomic DNA for each sample were determined using a NanoDrop Spectrophotometer (Thermo Scientific, Wilmington, DE). If the extracted DNA did not meet the experiment's concentration requirement (at least 50 ng/μl), it was re-extracted. The quality of the extracted DNA was assessed by agarose gel electrophoresis (Tanon, Shanghai, China). A single, clear main band with a faint side band indicated that the extracted DNA quality was high and uncontaminated. Primer Premier 5 (Premier Biosoft, CA) primer design software was used to design primers targeting a ∼100bp region around the polymorphic loci, and primer sequences were compared in the NCBI database to determine specificity. The primers used for PCR amplification of the 15 SNPs, synthesized by Sangon Biotech Co., Ltd (Shanghai, China), are shown in Table 7. A PCR instrument (Eppendorf, DE) was used to amplify the sequence of the detection region of each polymorphic locus, which was called target fragment. The quality of the amplified target fragment was assessed by PAGE gel electrophoresis (Tanon, Shanghai, China). Individually labeled magnetic beads were linked to each target fragment and then retrieved for high-throughput sequencing. The magnetic bead's label corresponding to each target fragment was annotated for proper identification. Finally, a PSTAR-II high-throughput sequencing instrument (HYK, Shenzhen, China) was used, according to the manufacturer's instructions, to detect the target fragments in order to analyze the corresponding polymorphic loci. The sequencing principle of the PSTAR-II was as follows: the sequencing primer was combined to the joint sequence of the sequencing sample by annealing based on the principle of base complementation. Then the fluorescent probe was combined with the sample under the action of the sequencing enzyme. The fluorescent signal emitted at specific excitation wavelengths was collected, and the base of the test site on the sample was determined by the type of fluorescent signal. Information on the sequencing primers is shown in Table 8.

**Table 6:** Numbering and information of the 15 SNPs

SNP No.	SNP	RS_ID	Ref_SNP
SNP-01	miR-146a	rs2910164	C>G
SNP-02	MDS1-EVI1	rs6774494	G>A
SNP-03	XPC	rs2228000	C>T
SNP-04	HCG9	rs3869062	A>G
SNP-05	HCG9	rs16896923	T>C
SNP-06	MMP2	rs243865	C>T
SNP-07	SPLUNC1	rs2752903	T>C
SNP-08	SPLUNC1	rs750064	A>G
SNP-09	HLA-F	rs3129055	T>C
SNP-10	GABBR1	rs2076483	T>C
SNP-11	GABBR1	rs29232	G>A
SNP-12	TP53	rs1042522	C>G
SNP-13	IL-10	rs1800896	A>G
SNP-14	MDM2	rs2279744	T>G
SNP-15	GSTM1	——	+/DEL

**Table 7:** Primers used for PCR amplification of candidate SNPs

No.	SNP	Primer no.	Primer sequence
1	miR-146a	NPC-SNP-01(184)F	AAAGCCGATGTGTATCCTCAG
		NPC-SNP-01(184)R	GCCTTCTGTCTCCAGTCTTCC
2	MDS1-EVI1	NPC-SNP-02(175)F	GACATCACCTGTTCACCCACTC
		NPC-SNP-02(175)R	CAAGCCAGGAATCAAAGATGAC
3	XPC	NPC-SNP-03(136)F	CAAAGGCTGGGTCCAAGAG
		NPC-SNP-03(136)R	TCTGCCTTCTCACCATCGC
4	HCG9	NPC-SNP-04(136)F	AAGGTGAGTGCTCCCTGATGG
		NPC-SNP-04(136)R	CCTGACCTTGTGATCTACCCG
5	HCG9	NPC-SNP-05(152)F	TTGCTAACAACTATAAAAAAGTCGG
		NPC-SNP-05(152)R	GTCTCCTCCTTGGTATTCCATTC
6	MMP2	NPC-SNP-06(137)F	TTGCTGTTTTTCATCTCTGGGC
		NPC-SNP-06(137)R	GACAATCAAGGAAGGCTTCCTG
7	SPLUNC1	NPC-SNP-07(155)F	AAACAGGAGACCCTTGCCC
		NPC-SNP-07(155)R	GGAAGGGACCTCAGTCTCATC
8	SPLUNC1	NPC-SNP-08(132)F	TTCAGGGCTCTCCAAAGTAAA
		NPC-SNP-08(132)R	CCGCTCAGGAAACCATAAAGT
9	HLA-F	NPC-SNP-09(118)F	GTTGACACTGACGTTTCCTCC
		NPC-SNP-09(118)R	AATGGTTTGGGGGTGGAT
10	GABBR1	NPC-SNP-10(178)F	TGGCTTTTCTTGTTCTGTTTTG
		NPC-SNP-10(178)R	GGAAGAAGCACAATTGGGATAG
11	GABBR1	NPC-SNP-11(147)F	TGCTCTACTCCTATTCCCGATG
		NPC-SNP-11(147)R	AGCATTAGACCTGGCAAGACC
12	TP53	NPC-SNP-12(187)F	AAGCAATGGATGATTTGATGC
		NPC-SNP-12(187)R	TGGGAAGGGACAGAAGATGAC
13	IL-10	NPC-SNP-13(187)F	CCAACTGGCTCCCCTTACC
		NPC-SNP-13(187)R	GAAATAACAAGGAAAAGAAGTCAGG
14	MDM2	NPC-SNP-14(96)F	GCGGGAGTTCAGGGTAAAGG
		NPC-SNP-14(96)R	CTAGTGACCCGACAGGCACC
15	GSTM1	NPC-SNP-15(215)F	GAACTCCCTGAAAAGCTAAAGC
		NPC-SNP-15(215)R	CTTGGGCTCAAATATACGGTGG

**Table 8:** Sequencing primers of the 15 SNPs

No.	SNP	Primer no.	Primer sequence
1	miR-146a	NPC-SNP-ANCHOR-01	GGTCTGACACTGAC
2	MDS1-EVI1	NPC-SNP-ANCHOR-02	AGCCATGGCCTGTA
3	XPC	NPC-SNP-ANCHOR-03	TGGCAAGCTTGGGT
4	HCG9	NPC-SNP-ANCHOR-04	TTTTATTATGGGTT
5	HCG9	NPC-SNP-ANCHOR-05	CAAATCCTTTCACA
6	MMP2	NPC-SNP-ANCHOR-06	AGTGCTGGGTGGGG
7	SPLUNC1	NPC-SNP-ANCHOR-07	CCTACATCTGGGGT
8	SPLUNC1	NPC-SNP-ANCHOR-08	ACCAAATAGCTTAA
9	HLA-F	NPC-SNP-ANCHOR-09	AGCCAAAATCCAGA
10	GABBR1	NPC-SNP-ANCHOR-10	TGGGGAGGAGGGAA
11	GABBR1	NPC-SNP-ANCHOR-11	TTTTGATAGCATTG
12	TP53	NPC-SNP-ANCHOR-12	GGGAGCAGCCTCT
13	IL-10	NPC-SNP-ANCHOR-13	CCCAAAGAAGCCTT
14	MDM2	NPC-SNP-ANCHOR-14	GCGGCCCCGCAGC
15	GSTM1	NPC-SNP-ANCHOR-15	ATGCTCTGGAAAACT

### Statistical analysis

PSTAR-II v33.211 software was used for PSTAR high-throughput data analysis. After HWE test (http://www.oege.org/software/hardy-weinberg.html), the genotype frequencies between Group A and Group B were compared by two-sided χ^2^ test. Unconditional logistic regression analysis was used for estimating OR and 95% CI. Three genotypes were discriminated for each SNP (except for GSTM1), and the homozygous wild-type was used as the reference for statistical analysis. A P-value <0.05 was considered statistically significant. Statistical analysis was performed using SPSS version 19.0 software (SPSS Inc., Chicago, IL, USA).

## SUPPLEMENTARY MATERIALS FIGURES AND TABLES





## Abbreviations

NPC: nasopharyngeal carcinoma
EBV: Epstein-Barr virus
SNP: single nucleotide polymorphism
PCR: polymerase chain reaction
NGS: next-generation sequencing technology
HWE: Hardy-Weinberg equilibrium
GSTM1: Glutathione S-transferase M1
IL-10: Interleukin-10
GABBR1: Gamma aminobutyric acid b receptor 1
MDM2: Murine double minute 2
miR-146a: Micro ribonucleic acid-146a
MDS1-EVI1: Myelodysplasia 1 and ecotropic viral insertion site 1 fusion proteins
XPC: Xeroderma pigmentosum group C
NER: nucleotide excision repair
HLA: human leukocyte antigen
MMP2: Matrix metalloproteinase 2
SPLUNC1: Short palate lung and nasal epithelium clone 1
OR: odds ratio
CI: confidence interval
