# Supplementary material for: Detection of nasopharyngeal carcinoma susceptibility with single nucleotide polymorphism analysis using next-generation sequencing technology
Source: Oncotarget. 2017 Apr 13;8(32):52708–23. doi: 10.18632/oncotarget.17085 (PMC5581063; doi:10.18632/oncotarget.17085)
Supplement: Supplementary file 1 [file oncotarget-08-52708-s001.pdf]

Detection of nasopharyngeal carcinoma susceptibility with single nucleotide polymorphism analysis using next-generation sequencing technology

SUPPLEMENTARY MATERIALS

Supplementary Table 1: Characteristics of eligible studies on TP53 (rs1042522, C>G).

See Supplementary File 1

Supplementary Table 2: Characteristics of eligible studies on GSTM1(+/DEL)

First author [Reference]	Year	Country(City)	Genotyping method	Conclusion (positive/ negative)	No.(cases/ controls)	Genotypes case		Genotypes control		Quality scores
						null	non-null	null	non-null	
Cheng YJ [19]	2003	China(Taiwan)	PCR	negative	314/337	173	141	169	168	13
Guo XC [20]	2008	China(Guangxi)	PCR	negative	341/590	204	137	328	262	13
Jiang Y [21]	2011	China(Qingddao)	PCR-CTPP	positive	182/372	97	85	157	215	11

Supplementary Table 3: Characteristics of eligible studies on IL-10 (rs1800896, A>G).

See Supplementary File 1

Supplementary Table 4: Characteristics of eligible studies on GABBR1 (rs2076483, T>C).

See Supplementary File 1

Supplementary Table 5: Characteristics of eligible studies on MDM2 (rs2279744, T>G).

See Supplementary File 1

Supplementary Table 6: Characteristics of eligible studies on miR-146a (rs2910164, C>G).

See Supplementary File 1

Supplementary Table 7: Characteristics of eligible studies on MDS1-EVI1 (rs6774494, G>A).

See Supplementary File 1

Supplementary Table 8: Characteristics of eligible studies on XPC (rs2228000, C>T).

See Supplementary File 1

Supplementary Table 9: Characteristics of eligible studies on GABBR1 (rs29232, G>A).

See Supplementary File 1

Supplementary Table 10: Characteristics of eligible studies on HCG9 (rs3869062, A>G).

See Supplementary File 1

Supplementary Table 11: Characteristics of eligible studies on HLA-F (rs3129055, T>C).

See Supplementary File 1

Supplementary Table 12: Characteristics of eligible studies on HCG9 (rs16896923, T>C).

See Supplementary File 1

Supplementary Table 13: Characteristics of eligible studies on MMP2 (rs243865, C>T).

See Supplementary File 1

Supplementary Table 14: Characteristics of eligible studies on SPLUNC1 (rs2752903, T>C).

See Supplementary File 1

Supplementary Table 15: Characteristics of eligible studies on SPLUNC1 (rs750064, A>G).

See Supplementary File 1

Supplementary Table 16: Scale for quality assessment

Criteria	Score
Representativeness of cases	
Selected from population or cancer registry	3
Selected from hospital	2
Selected from pathology archives, but without description	1
Not described	0
Credibility of controls	
Population-based	3
Blood donors or volunteers	2
Hospital-based (cancer-free patients)	1
Not described	0
Specimens of cases determining genotypes	
White blood cells or normal tissues	3
Tumor tissues or exfoliated cells of tissue	0
Hardy-Weinberg equilibrium in controls	
Hardy-Weinberg equilibrium	3
Hardy-Weinberg disequilibrium	0
Total sample size	
≥1000	3
≥400 but <1000	2
≥200 but <400	1
<200	0
