# Supplementary material for: Detection of nasopharyngeal carcinoma susceptibility with single nucleotide polymorphism analysis using next-generation sequencing technology
Source: Oncotarget. 2017 Apr 13;8(32):52708–23. doi: 10.18632/oncotarget.17085 (PMC5581063; doi:10.18632/oncotarget.17085)
Supplement: Supplementary file 2 [file oncotarget-08-52708-s002.docx]

| **Supplementary Table 1: Characteristics of eligible studies on TP53 (rs1042522, C>G).** | | | | | | | | | | | | | | | | | | | | | |
| --- | --- | --- | --- | --- | --- | --- | --- | --- | --- | --- | --- | --- | --- | --- | --- | --- | --- | --- | --- | --- | --- |
| **First author**  **[Reference]** | **Year** | **Country(City)** | **Genotyping**  **method** | **Conclusion (positive /negative)** | **No.(cases**  **/controls)** | **Genotypes case** | | | | | | | **Genotypes control** | | | | | | | **HWEP values (cases**  **/controls)** | **Quality**  **scores** |
|  |  |  |  |  |  | **C(Pro)** | **G(Arg)** | **CC** | **CG** | **GG** | **CC+CG** | **CG+GG** | **C(Pro)** | **G(Arg)** | **CC** | **CG** | **GG** | **CC+CG** | **CG+GG** |  |  |
| Golovleva I [14] | 1997 | China(Guizhou) | PCR | negative | 64/99 | 72 | 56 | 23 | 26 | 15 | 49 | 41 | 92 | 106 | 23 | 46 | 30 | 69 | 76 | 0.162/0.511 | 10 |
| Tsai MH [15] | 2002 | China(Taiwan) | PCR | positive | 50/59 | 46 | 54 | 16 | 14 | 20 | 30 | 34 | 42 | 76 | 8 | 26 | 25 | 34 | 51 | 0.002/0.765 | 9 |
| Xiao M [16] | 2010 | China(Hangzhou) | PCR-RFLP | positive | 522/712 | 540 | 504 | 135 | 270 | 117 | 405 | 387 | 606 | 818 | 120 | 366 | 226 | 486 | 592 | 0.415/0.170 | 14 |
| Li LJ [17] | 2013 | China(Chongqing) | PCR-RFLP | negative | 217/360 | 175 | 259 | 31 | 113 | 73 | 144 | 186 | 284 | 436 | 49 | 186 | 125 | 235 | 311 | 0.227/0.122 | 13 |
| Zhang XA [18] | 2014 | China(Nanning) | PCR | negative | 566/477 | 574 | 558 | 141 | 292 | 133 | 433 | 425 | 465 | 489 | 118 | 229 | 130 | 347 | 359 | 0.446/0.392 | 14 |

| **Supplementary Table 3: Characteristics of eligible studies on IL-10 (rs1800896, A>G).** | | | | | | | | | | | | | | | | | | | | | |
| --- | --- | --- | --- | --- | --- | --- | --- | --- | --- | --- | --- | --- | --- | --- | --- | --- | --- | --- | --- | --- | --- |
| **First author**  **[Reference]** | **Year** | **Country(City)** | **Genotyping**  **method** | **Conclusion (positive /negative)** | **No.(cases**  **/controls)** | **Genotypes case** | | | | | | | **Genotypes control** | | | | | | | **HWEP values (cases**  **/controls)** | **Quality**  **scores** |
|  |  |  |  |  |  | **A** | **G** | **AA** | **AG** | **GG** | **AA+AG** | **AG+GG** | **A** | **G** | **AA** | **AG** | **GG** | **AA+AG** | **AG+GG** |  |  |
| Tsai CW [22] | 2013 | China(Taiwan) | PCR-RFLP | positive | 176/522 | 283 | 69 | 117 | 49 | 10 | 166 | 59 | 930 | 114 | 419 | 92 | 11 | 511 | 103 | 0.122/0.032 | 8 |
| Wei YS [23] | 2007 | China(Sichuan) | PCR-RFLP | positive | 198/210 | 307 | 89 | 123 | 61 | 14 | 184 | 75 | 372 | 48 | 167 | 38 | 5 | 205 | 43 | 0.103/0.124 | 12 |

| **Supplementary Table 4: Characteristics of eligible studies on GABBR1 (rs2076483, T>C).** | | | | | | | | | | | | | | | | | | | | | |
| --- | --- | --- | --- | --- | --- | --- | --- | --- | --- | --- | --- | --- | --- | --- | --- | --- | --- | --- | --- | --- | --- |
| **First author**  **[Reference]** | **Year** | **Country(City)** | **Genotyping**  **method** | **Conclusion (positive /negative)** | **No.(cases**  **/controls)** | **Genotypes case** | | | | | | | **Genotypes control** | | | | | | | **HWEP values (cases**  **/controls)** | **Quality**  **scores** |
|  |  |  |  |  |  | **T** | **C** | **TT** | **TC** | **CC** | **TT+TC** | **TC+CC** | **T** | **C** | **TT** | **TC** | **CC** | **TT+TC** | **TC+CC** |  |  |
| Hsu WL [24] | 2012 | China(Taiwan) | PCR | positive | 586/521 | 972 | 200 | 403 | 166 | 17 | 569 | 183 | 759 | 283 | 273 | 213 | 35 | 486 | 248 | 0.985/0.447 | 14 |
| Tse KP [25] | 2009 | China(Taiwan) | PCR | positive | 912/1925 | 1498 | 326 | - | - | - | - | - | 2853 | 997 | - | - | - | - | - | >0.05 | 14 |

| **Supplementary Table 5: Characteristics of eligible studies on MDM2 (rs2279744, T>G).** | | | | | | | | | | | | | | | | | | | | | |
| --- | --- | --- | --- | --- | --- | --- | --- | --- | --- | --- | --- | --- | --- | --- | --- | --- | --- | --- | --- | --- | --- |
| **First author**  **[Reference]** | **Year** | **Country(City)** | **Genotyping**  **method** | **Conclusion (positive /negative)** | **No.(cases**  **/controls)** | **Genotypes case** | | | | | | | **Genotypes control** | | | | | | | **HWEP values (cases**  **/controls)** | **Quality**  **scores** |
|  |  |  |  |  |  | **T** | **G** | **TT** | **TG** | **GG** | **TT+TG** | **TG+GG** | **T** | **G** | **TT** | **TG** | **GG** | **TT+TG** | **TG+GG** |  |  |
| Xiao M [16] | 2010 | China(Hangzhou) | PCR-RFLP | positive | 522/712 | 465 | 579 | 111 | 243 | 168 | 354 | 411 | 822 | 602 | 238 | 346 | 128 | 584 | 474 | 0.187/0.908 | 14 |
| Zhou G [26] | 2007 | China(Guangxi+Guangdong) | PCR | positive | 803/763 | 676 | 930 | 151 | 374 | 278 | 525 | 652 | 730 | 796 | 191 | 348 | 224 | 539 | 572 | 0.206/0.017 | 11 |

| **Supplementary Table 6: Characteristics of eligible studies on miR-146a (rs2910164, C>G).** | | | | | | | | | | | | | | | | | | | | | |
| --- | --- | --- | --- | --- | --- | --- | --- | --- | --- | --- | --- | --- | --- | --- | --- | --- | --- | --- | --- | --- | --- |
| **First author**  **[Reference]** | **Year** | **Country(City)** | **Genotyping**  **method** | **Conclusion (positive /negative)** | **No.(cases**  **/controls)** | **Genotypes case** | | | | | | | **Genotypes control** | | | | | | | **HWEP values (cases**  **/controls)** | **Quality**  **scores** |
|  |  |  |  |  |  | **C** | **G** | **CC** | **CG** | **GG** | **CC+CG** | **CG+GG** | **C** | **G** | **CC** | **CG** | **GG** | **CC+CG** | **CG+GG** |  |  |
| Huang GL [27] | 2014 | China(Changsha) | PCR-RFLP | positive | 160/200 | 201 | 119 | 64 | 73 | 23 | 137 | 96 | 218 | 182 | 54 | 110 | 36 | 164 | 146 | 0.768/0.123 | 10 |
| Lung RW [28] | 2013 | China(HongKong) | PCR | positive | 229/3776 | 322 | 136 | 117 | 88 | 24 | 205 | 112 | 4751 | 2801 | 1472 | 1807 | 497 | 3279 | 2304 | 0.228/0.118 | 13 |

| **Supplementary Table 7: Characteristics of eligible studies on MDS1-EVI1 (rs6774494, G>A).** | | | | | | | | | | | | | | | | | | | | | |
| --- | --- | --- | --- | --- | --- | --- | --- | --- | --- | --- | --- | --- | --- | --- | --- | --- | --- | --- | --- | --- | --- |
| **First author**  **[Reference]** | **Year** | **Country(City)** | **Genotyping**  **method** | **Conclusion (positive /negative)** | **No.(cases**  **/controls)** | **Genotypes case** | | | | | | | **Genotypes control** | | | | | | | **HWEP values (cases**  **/controls)** | **Quality**  **scores** |
|  |  |  |  |  |  | **G** | **A** | **GG** | **GA** | **AA** | **GG+GA** | **GA+AA** | **G** | **A** | **GG** | **GA** | **AA** | **GG+GA** | **GA+AA** |  |  |
| Bei JX [29] | 2010 | China(Guangdong) | PCR | positive | 5090/4957 | 3257 | 6923 | - | - | - | - | - | 5023 | 4891 | - | - | - | - | - | >0.05 | 14 |
| Yee Ko JM [30] | 2014 | China(Hong Kong) | PCR | positive | 1177/1172 | 716 | 1638 | - | - | - | - | - | 841 | 1503 | - | - | - | - | - | >0.05 | 13 |

| **Supplementary Table 8: Characteristics of eligible studies on XPC (rs2228000, C>T).** | | | | | | | | | | | | | | | | | | | | | |
| --- | --- | --- | --- | --- | --- | --- | --- | --- | --- | --- | --- | --- | --- | --- | --- | --- | --- | --- | --- | --- | --- |
| **First author**  **[Reference]** | **Year** | **Country(City)** | **Genotyping**  **method** | **Conclusion (positive /negative)** | **No.(cases**  **/controls)** | **Genotypes case** | | | | | | | **Genotypes control** | | | | | | | **HWEP values (cases**  **/controls)** | **Quality**  **scores** |
|  |  |  |  |  |  | **C** | **T** | **CC** | **CT** | **TT** | **CC+CT** | **CT+TT** | **C** | **T** | **CC** | **CT** | **TT** | **CC+CT** | **CT+TT** |  |  |
| Yee Ko JM [30] | 2014 | China(Hong Kong) | PCR | positive | 1177/1172 | 1358 | 996 | - | - | - | - | - | 1432 | 912 | - | - | - | - | - | >0.05 | 13 |
| Yang ZH [31] | 2008 | China(Sichuan) | PCR-RFLP | positive | 153/168 | 177 | 129 | 52 | 73 | 28 | 125 | 101 | 231 | 105 | 76 | 79 | 13 | 155 | 92 | 0.789/0.221 | 11 |

| **Supplementary Table 9: Characteristics of eligible studies on GABBR1 (rs29232, G>A).** | | | | | | | | | | | | | | | | | | | | | |
| --- | --- | --- | --- | --- | --- | --- | --- | --- | --- | --- | --- | --- | --- | --- | --- | --- | --- | --- | --- | --- | --- |
| **First author**  **[Reference]** | **Year** | **Country(City)** | **Genotyping**  **method** | **Conclusion (positive /negative)** | **No.(cases**  **/controls)** | **Genotypes case** | | | | | | | **Genotypes control** | | | | | | | **HWEP values (cases**  **/controls)** | **Quality**  **scores** |
|  |  |  |  |  |  | **G** | **A** | **GG** | **GA** | **AA** | **GG+GA** | **GA+AA** | **G** | **A** | **GG** | **GA** | **AA** | **GG+GA** | **GA+AA** |  |  |
| Hsu WL [24] | 2012 | China(Taiwan) | PCR | positive | 586/517 | 518 | 654 | 114 | 290 | 182 | 404 | 472 | 601 | 433 | 171 | 259 | 87 | 430 | 346 | 0.937/0.508 | 14 |
| Tse KP [25] | 2009 | China(Taiwan) | PCR | positive | 912/1925 | 755 | 1069 | - | - | - | - | - | 2094 | 1756 | - | - | - | - | - | >0.05 | 14 |

| **Supplementary Table 10: Characteristics of eligible studies on HCG9 (rs3869062, A>G).** | | | | | | | | | | | | | | | | | | | | | |
| --- | --- | --- | --- | --- | --- | --- | --- | --- | --- | --- | --- | --- | --- | --- | --- | --- | --- | --- | --- | --- | --- |
| **First author**  **[Reference]** | **Year** | **Country(City)** | **Genotyping**  **method** | **Conclusion (positive /negative)** | **No.(cases**  **/controls)** | **Genotypes case** | | | | | | | **Genotypes control** | | | | | | | **HWEP values (cases**  **/controls)** | **Quality**  **scores** |
|  |  |  |  |  |  | **A** | **G** | **AA** | **AG** | **GG** | **AA+AG** | **AG+GG** | **A** | **G** | **AA** | **AG** | **GG** | **AA+AG** | **AG+GG** |  |  |
| Hsu WL [24] | 2012 | China(Taiwan) | PCR | positive | 572/507 | 893 | 251 | 343 | 207 | 22 | 550 | 229 | 678 | 336 | 223 | 232 | 52 | 455 | 284 | 0.177/0.462 | 14 |
| Tse KP [25] | 2009 | China(Taiwan) | PCR | positive | 912/1925 | 1445 | 379 | - | - | - | - | - | 2630 | 1220 | - | - | - | - | - | >0.05 | 14 |

| **Supplementary Table 11: Characteristics of eligible studies on HLA-F (rs3129055, T>C).** | | | | | | | | | | | | | | | | | | | | | |
| --- | --- | --- | --- | --- | --- | --- | --- | --- | --- | --- | --- | --- | --- | --- | --- | --- | --- | --- | --- | --- | --- |
| **First author**  **[Reference]** | **Year** | **Country(City)** | **Genotyping**  **method** | **Conclusion (positive /negative)** | **No.(cases**  **/controls)** | **Genotypes case** | | | | | | | **Genotypes control** | | | | | | | **HWEP values (cases**  **/controls)** | **Quality**  **scores** |
|  |  |  |  |  |  | **T** | **C** | **TT** | **TC** | **CC** | **TT+TC** | **TC+CC** | **T** | **C** | **TT** | **TC** | **CC** | **TT+TC** | **TC+CC** |  |  |
| Hsu WL [24] | 2012 | China(Taiwan) | PCR | positive | 588/520 | 704 | 472 | 222 | 260 | 106 | 482 | 366 | 723 | 317 | 246 | 231 | 43 | 477 | 274 | 0.053/0.272 | 14 |
| Tse KP [25] | 2009 | China(Taiwan) | PCR | positive | 912/1925 | 1083 | 741 | - | - | - | - | - | 2649 | 1201 | - | - | - | - | - | >0.05 | 14 |

| **Supplementary Table 12: Characteristics of eligible studies on HCG9 (rs16896923, T>C).** | | | | | | | | | | | | | | | | | | | | | |
| --- | --- | --- | --- | --- | --- | --- | --- | --- | --- | --- | --- | --- | --- | --- | --- | --- | --- | --- | --- | --- | --- |
| **First author**  **[Reference]** | **Year** | **Country(City)** | **Genotyping**  **method** | **Conclusion (positive /negative)** | **No.(cases**  **/controls)** | **Genotypes case** | | | | | | | **Genotypes control** | | | | | | | **HWEP values (cases**  **/controls)** | **Quality**  **scores** |
|  |  |  |  |  |  | **T** | **C** | **TT** | **TC** | **CC** | **TT+TC** | **TC+CC** | **T** | **C** | **TT** | **TC** | **CC** | **TT+TC** | **TC+CC** |  |  |
| Hsu WL [24] | 2012 | China(Taiwan) | PCR | positive | 582/514 | 989 | 175 | 413 | 163 | 6 | 576 | 169 | 779 | 249 | 292 | 195 | 27 | 487 | 222 | 0.020/0.448 | 14 |
| Tse KP [25] | 2009 | China(Taiwan) | PCR | positive | 912/1925 | 1550 | 274 | - | - | - | - | - | 2957 | 893 | - | - | - | - | - | >0.05 | 14 |

| **Supplementary Table 13: Characteristics of eligible studies on MMP2 (rs243865, C>T).** | | | | | | | | | | | | | | | | | | | | | |
| --- | --- | --- | --- | --- | --- | --- | --- | --- | --- | --- | --- | --- | --- | --- | --- | --- | --- | --- | --- | --- | --- |
| **First author**  **[Reference]** | **Year** | **Country(City)** | **Genotyping**  **method** | **Conclusion (positive /negative)** | **No.(cases**  **/controls)** | **Genotypes case** | | | | | | | **Genotypes control** | | | | | | | **HWEP values (cases**  **/controls)** | **Quality**  **scores** |
|  |  |  |  |  |  | **C** | **T** | **CC** | **CT** | **TT** | **CC+CT** | **CT+TT** | **C** | **T** | **CC** | **CT** | **TT** | **CC+CT** | **CT+TT** |  |  |
| Shao JY [32] | 2011 | China(Guangdong) | PCR | positive | 370/390 | 679 | 61 | 311 | 57 | 2 | 368 | 59 | 692 | 88 | 306 | 80 | 4 | 386 | 84 | 0.724/0.626 | 13 |
| Zhou G [33] | 2007 | China(Guangxi+Guangdong) | PCR | positive | 803/759 | 1539 | 67 | 736 | 67 | 0 | 803 | 67 | 1404 | 114 | 646 | 112 | 1 | 758 | 113 | 0.217/0.086 | 13 |

| **Supplementary Table 14: Characteristics of eligible studies on SPLUNC1 (rs2752903, T>C).** | | | | | | | | | | | | | | | | | | | | | |
| --- | --- | --- | --- | --- | --- | --- | --- | --- | --- | --- | --- | --- | --- | --- | --- | --- | --- | --- | --- | --- | --- |
| **First author**  **[Reference]** | **Year** | **Country(City)** | **Genotyping**  **method** | **Conclusion (positive /negative)** | **No.(cases**  **/controls)** | **Genotypes case** | | | | | | | **Genotypes control** | | | | | | | **HWEP values (cases**  **/controls)** | **Quality**  **scores** |
|  |  |  |  |  |  | **T** | **C** | **TT** | **TC** | **CC** | **TT+TC** | **TC+CC** | **T** | **C** | **TT** | **TC** | **CC** | **TT+TC** | **TC+CC** |  |  |
| Yew PY [34] | 2012 | Malaysia(Malaysian Chinese) | PCR | positive | 445/487 | 643 | 247 | 233 | 177 | 35 | 410 | 212 | 768 | 206 | 312 | 144 | 31 | 456 | 175 | 0.864/0.012 | 9 |
| He Y [35] | 2005 | China(Guangzhou) | PCR | positive | 239/281 | 290 | 188 | 120 | 50 | 69 | 170 | 119 | 401 | 161 | 160 | 81 | 40 | 241 | 121 | 0.000/0.000 | 9 |

| **Supplementary Table 15: Characteristics of eligible studies on SPLUNC1 (rs750064, A>G).** | | | | | | | | | | | | | | | | | | | | | |
| --- | --- | --- | --- | --- | --- | --- | --- | --- | --- | --- | --- | --- | --- | --- | --- | --- | --- | --- | --- | --- | --- |
| **First author**  **[Reference]** | **Year** | **Country(City)** | **Genotyping**  **method** | **Conclusion (positive /negative)** | **No.(cases**  **/controls)** | **Genotypes case** | | | | | | | **Genotypes control** | | | | | | | **HWEP values (cases**  **/controls)** | **Quality**  **scores** |
|  |  |  |  |  |  | **A** | **G** | **AA** | **AG** | **GG** | **AA+AG** | **AG+GG** | **A** | **G** | **AA** | **AG** | **GG** | **AA+AG** | **AG+GG** |  |  |
| Yew PY [34] | 2012 | Malaysia(Malaysian Chinese) | PCR | negative | 81/147 | 86 | 76 | 24 | 38 | 19 | 62 | 57 | 175 | 119 | 52 | 71 | 24 | 123 | 95 | 0.601/0.977 | 11 |
| He Y [35] | 2005 | China(Guangzhou) | PCR | positive | 232/282 | 249 | 215 | 64 | 121 | 47 | 185 | 168 | 361 | 203 | 99 | 163 | 20 | 262 | 183 | 0.458/0.000 | 9 |
